# AC Electrokinetics-Enhanced Capacitive Aptasensor for Point-of-Care Testing of Acrylamide in Coffee

**DOI:** 10.3390/mi17080966

**Published:** 2026-08-16

**Authors:** Ke Wang, Mingna Xie, Yuyang Zhao, Jiuyi Wang, Leilei Zeng, Xiaogang Lin, Jie Jayne Wu

**Affiliations:** 1 Key Laboratory of Optoelectronic Technology and Systems, Ministry of Education, Chongqing University, Chongqing, 400044, China; cqu_wke@163.com (K.W.); mingnaxie2017cqu@163.com (M.X.); 202508131179@stu.cqu.edu.cn (Y.Z.); 202408021039t@stu.cqu.edu.cn (J.W.); zengll0310@163.com (L.Z.); 2 Department of Electrical Engineering and Computer Science, The University of Tennessee, Knoxville, TN 37996, USA; jaynewu@utk.edu

**Keywords:** acrylamide, capacitive sensor, alternating current electrokinetics effect, aptamer, point-of-care testing

## Abstract

Acrylamide (AA) is a common contaminant in foods processed at high temperatures and has attracted significant attention due to its potential neurotoxicity and carcinogenicity. Therefore, the development of a highly sensitive, highly selective sensing technology suitable for on-site detection is of great importance for ensuring food safety. In this study, an aptamer (Apt)-based capacitive AA sensor was developed based on the alternating current electrokinetics (ACEK) effect. The sensor utilizes an aptamer as the biomimetic recognition element, which can specifically recognize AA, thereby enabling quantitative detection. Additionally, a detachable detection fixture and data acquisition system were designed to enhance the detection stability and convenience of sensor. Within the linear range of 1 nmol/L to 10 µmol/L, the sensor response (dC/dt) exhibited a good linear relationship with AA concentration, with a detection limit as low as 0.4235 nmol/L. The sensor exhibits good selectivity toward structural analogs of AA, with a recovery relative standard deviations (RSDs) of less than 5.42% in spiked coffee samples. This portable detection system provides a sensitive and user-friendly tool for the analysis of acrylamide in food and holds great potential for application in food safety.

## 1. Introduction

Acrylamide is a colorless and odorless small organic molecule that is typically formed during food processing through two major mechanisms: the Strecker degradation pathway and the acrolein pathway [1,2]. Consequently, AA can be generated not only via the Strecker pathway in carbohydrate-rich foods such as potato products and bread, but also through the acrolein pathway in lipid-rich foods such as cured black olives [3]. Extensive studies have demonstrated that AA possesses potential carcinogenic, neurotoxic, and genotoxic effects, as well as a high capacity to permeate the reproductive system [4,5]. Many countries and international organizations have conducted risk assessments and established maximum residue limits for AA.

Traditional analytical techniques such as gas chromatography–mass spectrometry (GC–MS) [6], liquid chromatography–tandem mass spectrometry (LC–MS/MS) [7,8,9], high-performance liquid chromatography (HPLC), capillary electrophoresis (CE) [10], and enzyme-linked immunosorbent assay (ELISA) [11] offer high accuracy, sensitivity, and selectivity, but these methods still face several inherent limitations, including high equipment cost, complex operation, stringent environmental requirements, tedious sample pretreatment, and time-consuming analysis procedures. To address these issues, emerging sensor technologies based on nanomaterials [12,13,14], surface-enhanced Raman scattering (SERS) assays [15,16,17], fluorescent probes [18,19], and electrochemical assays [20,21,22] have drawn increasing attention. Achieving on-site and rapid detection of AA is of great significance for improving both the efficiency and accessibility of food safety monitoring. However, current AA detection methods and sensor systems still exhibit some shortcomings. The preparation and characterization of nanomaterials are time-consuming and complex. SERS detection methods rely on SERS labels and the uniform distribution of hot spots on the substrate. In addition, generating a strong SERS signal typically requires the use of nanomaterials, which complicates the initial preparation process. Although sensors based on quartz crystal microbalance (QCM) offer label-free and real-time detection capabilities, their detection stability requires improvement, and the activity of functionalized electrode layers tends to degrade under low-temperature storage conditions [23]. Similarly, fluorescence resonance energy transfer (FRET)-based nanoprobe sensing platforms can achieve ultralow detection limits, yet their applications remain limited by the high cost of fluorescent probe materials and insufficient anti-interference performance in complex food matrices [24,25].

Aptamers are unique single-stranded RNA or DNA oligonucleotides obtained through Systematic Evolution of Ligands by Exponential Enrichment (SELEX) [26]. Among various biorecognition elements, aptamers stand out owing to their small molecular size, ease of synthesis and modification, cost-effectiveness, nontoxicity, reusability, high thermal and chemical stability, and excellent binding specificity toward diverse targets [27]. Although aptamer-based colorimetric sensors [5,28] enable portable detection, the limited stability of immobilized aptamers restricts their long-term performance, and the detection time is often uncertain. Therefore, the development of a point-of-care testing (POCT) system for AA that combines high sensitivity, specificity, stability, and portability remains an urgent need.

In this study, we propose a capacitive sensing mechanism integrated with an ACEK-based enrichment strategy to design and fabricate a rapid-response, high-sensitivity microsensor. Furthermore, an instantaneous detection system was developed to enable real-time quantitative analysis of AA in complex food matrices. A self-assembled monolayer (SAM) of 11-mercaptoundecanoic acid (MUA) was employed to ensure a uniform and stable sensing interface, and the aptamer probes were immobilized on the surface through a carboxyl–amino covalent coupling strategy. Using a self-developed high-precision data acquisition module based on the auto-balancing bridge principle, the system continuously recorded interfacial capacitance values, and the rate of capacitance change within 1 min (dC/dt) was used to quantify AA concentrations. The proposed method demonstrated excellent selectivity toward AA, with spiked recovery rates of 93.96–104.63% and RSD below 5.42% in coffee matrix samples. This study presents a portable platform capable of simultaneously performing target binding and detection under AC stimulation, offering a new approach to rapid molecular detection.

## 2. Materials and Methods

### 2.1. Reagents and Materials

Ethanol (AR), methacrylamide (MAA), and L-asparagine (Asn) were purchased from Shanghai Aladdin Biochemical Technology Co., Ltd. (Shanghai, China). Acetone (AR), isopropanol (AR), ethanolamine hydrochloride (98%), and 1-(3-dimethylaminopropyl)-3-ethylcarbodiimide (EDC, 98.5%) were obtained from Shanghai Macklin Biochemical Co., Ltd. (Shanghai, China). N-hydroxysuccinimide (NHS, 99%), 11-mercaptoundecanoic acid (MUA, 95%), and phosphate-buffered saline (1 × PBS, PH 7.4) were purchased from Sigma-Aldrich (Shanghai) Trading Co., Ltd. (Shanghai, China), Shanghai Yuanye Bio-Technology Co., Ltd. (Shanghai, China), and Beijing Solarbio Science & Technology Co., Ltd. (Beijing, China), respectively. The amino-modified AA aptamer (5′-NH_2_-CAGTCCAGGACAGATTCGCGAGTGGTCGTGGTGAGGTGCGTGTATGGGTGGTGGATGAGTGTGTGGCCACGTGGATTTCATTCAGCGATT-3′) was synthesized and purified by Sangon Biotechnology Co., Ltd. (Shanghai, China) [5,29,30].

### 2.2. Apparatus and Characterization

Scanning electron microscopy (SEM) images were obtained using a Quattro S Scanning Electron Microscope (Thermo Fisher Scientific, Waltham, MA, USA). An ultrasonic cleaner (JP-020, Jiemeng Co., Shenzhen, China), ultraviolet ozone cleaner (PSD-UVT, Novascan, IL, USA), constant temperature and humidity incubator (HWS-50, Shaoxing Bowei Instrument Equipment Co., Ltd., Shaoxing, China), vortex mixer (MX-S, SCILOGEX, Rocky Hill, CT, USA), and mini centrifuge (L-CMP7, Labgic Co., Beijing, China) were employed for the treatment and incubation of functionalized sensor chips. A high-precision silicon-based two-electrode interdigitated gold electrode (Yuxin Co., Guangzhou, China) was used as the sensing substrate in this study. The dimensions of this electrode are 6 mm × 4 mm, with both the finger width and spacing of 5 μm, and contains 25 pairs of interdigitated fingers. All experimental procedures were performed under aseptic conditions in a clean bench (VD-650, Shanghai Shangdao Instrument Manufacturing Co., Ltd., Shanghai, China).

### 2.3. Functionalization of the Sensor Chip

**Figure 1:**
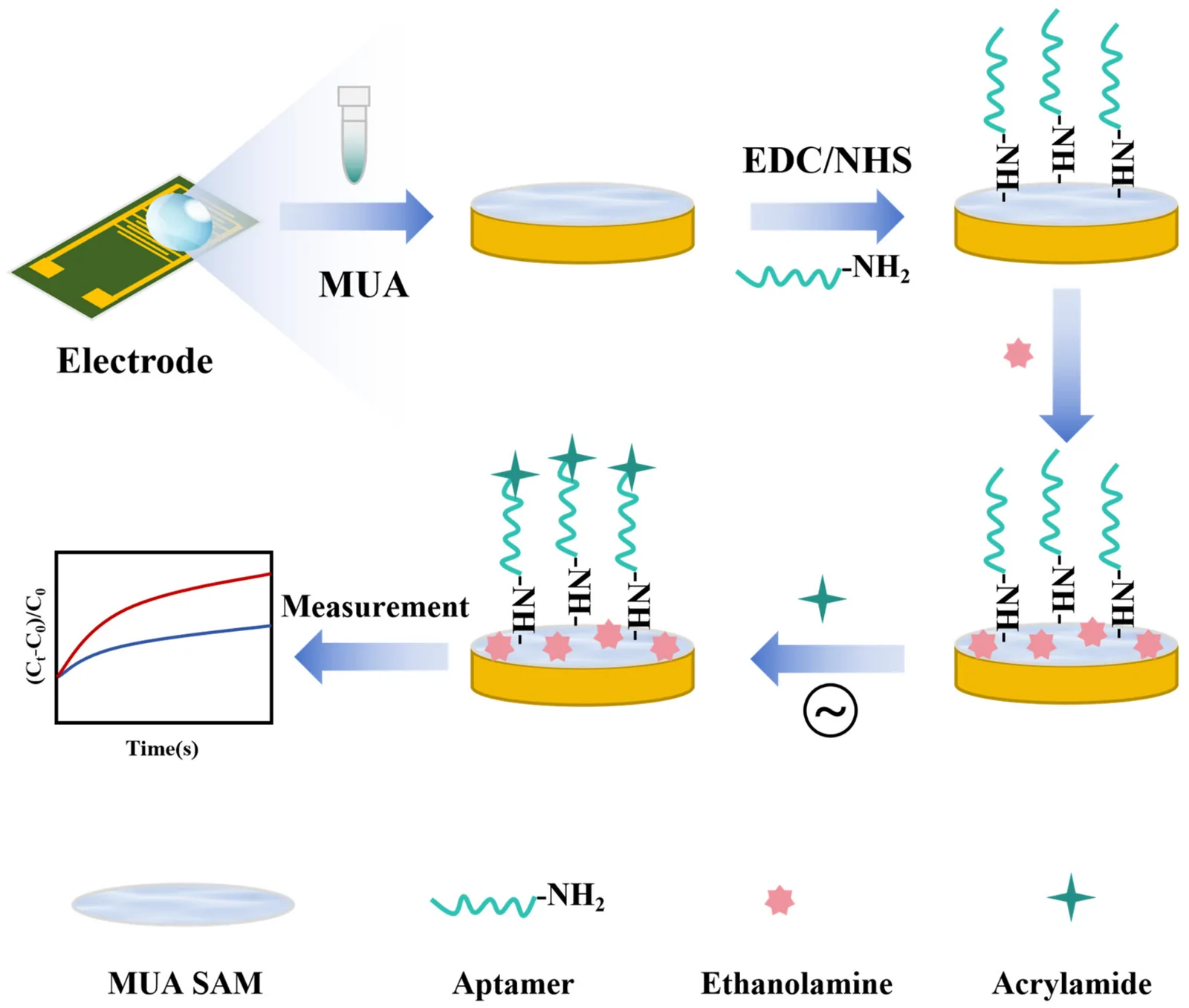
Schematic of the ACEK-enhanced capacitive aptasensor.

The functionalization process of the sensor chip is shown in Figure 1. To ensure effective surface functionalization, the electrodes were thoroughly cleaned with acetone and isopropyl alcohol. The chip was then rinsed sequentially with ultrapure water and anhydrous ethanol, and finally dried under nitrogen. The cleaned interdigitated electrodes were immersed in MUA solution and left to self-assemble at 4 °C for 24 h to form a stable MUA SAM (MUA/Au). The MUA/Au electrode was sequentially rinsed with anhydrous ethanol and ultrapure water, followed by drying under a nitrogen stream. Next, 10 μL of EDC/NHS activation solution was added dropwise, and the sample was activated at 25 °C for 1 h. Subsequently, another 2 μL of EDC/NHS activation solution was added dropwise, and activation was continued for 2 h to enhance the covalent bonding capacity between carboxyl and amino groups. Subsequently, 10 μL of aptamer solution (1.0 μM in 10 mM PBS, pH 7.4) was drop-cast and incubated at 25 °C for 2 h to yield the functionalized electrode (Apt/MUA/Au). Residual activated sites were blocked by adding ethanolamine hydrochloride solution (1 M, pH 8.5) and incubating at 25 °C for 1 h, thereby minimizing non-specific adsorption and improving detection stability and reproducibility. After blocking, excess blocking solution was removed with absorbent paper, and the electrode was thoroughly rinsed with ultrapure water. The functionalized electrode was now ready for subsequent AA detection experiments.

### 2.4. Design of a Detachable Detection Fixture

Due to the small size of the gold interdigitated electrode, the electrode surface is easily susceptible to contamination during both the incubation and testing stages. More importantly, during measurement, the electrode often suffers from poor contact, cannot be directly connected to the impedance analyzer, and repeated manual handling may lead to abrasion of the gold film surface. These issues compromise electrode stability and data reliability, and further hinder integration into the subsequent portable, on-site AA detection system. To address these challenges, a detachable detection fixture was designed and fabricated using SolidWorks 2024 for structural modeling and 3D printing technology for manufacturing. The fixture consists of two main components: an external base and an incubation chamber.

### 2.5. Design of the Sensor Data Acquisition Module

In this study, the normalized interfacial capacitance was adopted as the primary indicator to quantify changes at the sensor–electrolyte interface for samples with varying AA concentrations. To enable real-time monitoring of interfacial capacitance variations in the ACEK-enhanced capacitive aptasensor, a high-precision sensor data acquisition module was designed and implemented based on the auto-balancing bridge principle and digital phase-sensitive detection (PSD) technique. The proposed system adopts a dual-board architecture, consisting of a main control board and a functional circuit sub-board, interconnected via a board-to-board connector. This design ensures stable signal transmission and excellent system scalability. To further improve measurement precision, the module integrates four-terminal measurement, a virtual ground circuit, signal conditioning circuitry, and a high-precision excitation source. The incorporation of digital PSD in the firmware enhances the signal-to-noise ratio (SNR), enabling accurate impedance characterization.

### 2.6. Detection of AA Using the ACEK-Enhanced Aptasensor

The incubation chamber was assembled onto the main body of the detection fixture, ensuring stable electrical contact between the electrode and fixture. The test ports of the data acquisition module were connected via cables to the positive and negative terminals of the fixture to apply the AC signal. AA standard solutions (10 μL) at final concentrations of 10^−9^, 10^−8^, 10^−7^, 10^−6^, and 10^−5^ mol/L were individually introduced, and the interfacial capacitance change rate (dC/dt) was monitored over 1 min. Data were acquired using PC-based software and normalized. All measurements were performed in quintuplicate (*n* = 5).

Selectivity is a key factor determining the accuracy and reliability of a sensor. In addition to AA (the target analyte), Asn, acrylic acid (Aa), and MAA were selected as interferents. All analytes were tested at a concentration of 10 µmol/L, which falls within the linear dynamic range (1 nmol/L–10 µmol/L), to ensure that the response values fall within the sensitivity detection range of the sensor, thereby enabling accurate comparative analysis. To eliminate the effects of nonspecific adsorption or baseline drift, a blank control (containing only buffer) was included. Under the optimized ACEK parameters, five replicate measurements (*n* = 5) were performed for each test condition, and the capacitance transients over a 1 min period were recorded.

### 2.7. Application of the ACEK-Enhanced Aptasensor in Coffee Samples

Commercially available liquid coffee (Luckin Extra Strong Americano Cold Brew) was selected as the real sample matrix for spike-recovery experiments. The coffee was diluted 1:10 with ultrapure water to mimic typical consumption conditions, yielding the working coffee matrix. Spike samples were prepared at low (10 nmol/L), medium (1 μmol/L), and high (10 μmol/L) AA levels using this diluted matrix. Real sample (1 mL) or AA-spiked matrix was mixed with methanol (70% *v*/*v*, 10 mL), centrifuged, and filtered through a 0.45 μm syringe filter to achieve the target AA concentrations. AA content was quantified using the ACEK-enhanced aptasensor, and recovery rates were calculated.

## 3. Results and Discussion

### 3.1. Principle of the ACEK-Enhanced Aptasensor for AA Detection

For electrochemical measurements, the incubation chamber was securely integrated into the fixture assembly, with verified electrical continuity between the electrode and fixture contacts. An impedance analyzer was interfaced via shielded cables to the anode and cathode terminals to deliver the ACEK signal. The principle of ACEK enhancement is shown in Figure S1. In the absence of AA, the interfacial capacitance remained stable, exhibiting negligible drift. In the presence of AA, the ACEK effects dominated by the AC Electrothermal (ACET) and AC Electroosmosis (ACEO) effects can enhance the movement of AA in the medium [31,32,33]. The specific recognition of AA by the aptamer triggers conformational rearrangements and/or local dielectric perturbations at the electrode–electrolyte interface, resulting in changes in the sensing interface capacitance that can be measured in real time. The calculation of the rate of change in normalized interface capacitance (dC/dt) enables both qualitative identification and quantitative determination of AA across a dynamic range.

### 3.2. Characterization of Electrode Functionalization

**Figure 2:**
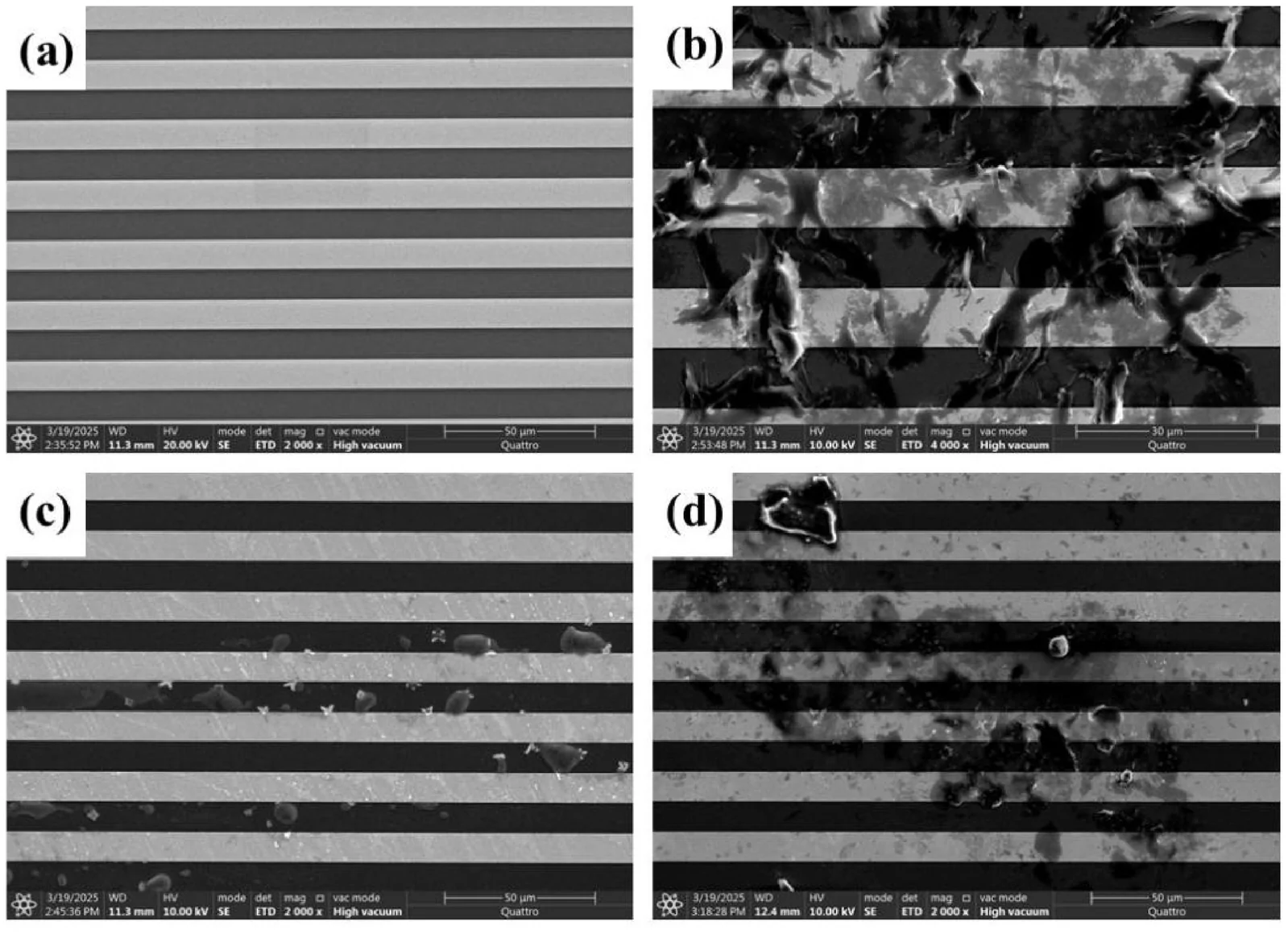
SEM images of (**a**) post-cleaning electrode, (**b**) Apt/MUA/Au, (**c**) Eth/Apt/MUA/Au, (**d**) AA@Apt/MUA/Au.

To evaluate surface morphology evolution and uniformity during stepwise functionalization, the electrodes were characterized using SEM coupled with energy-dispersive X-ray spectroscopy (EDS) to confirm elemental composition and verify modification efficacy. The surface morphology of the cleaned interdigitated gold electrode is shown in Figure 2a. The surface appears smooth and uniform, with no visible granular contaminants or residues. After MUA self-assembly was complete, the changes in surface morphology caused by this ultrathin monolayer were negligible. However, EDS detected a distinct sulfur (S) signal, confirming that the MUA SAM had been successfully formed (Figure S2a). As shown in Figure 2b, after covalent binding of the aptamer, slight roughening of the surface was observed. Localized cluster-like structures or aggregates also appeared, indicating an uneven distribution of the aptamer. At the same time, EDS detected nitrogen (N) originating from the aptamer’s main chain, providing indirect evidence of successful coupling (Figure S2b). To minimize nonspecific adsorption, excess active sites on the surface were blocked using ethanolamine hydrochloride (Eth). As shown in Figure 2c, the morphology of the blocked electrode surface was more uniform and flatter, with no unpassivated areas observed, indicating that the blocking treatment was effective. The EDS spectrum showed in Figure S2c revealed a decrease in nitrogen (N) content, confirming that unreacted EDC/NHS had been removed. At the same time, the chlorine (Cl) signal was enhanced, further verifying the success of the blocking process. When the aptamer specifically recognized AA, localized particle deposition and morphological disruption were observed in Figure 2d, indicating that target binding had occurred. EDS spectral analysis revealed enhanced signals for carbon (C), oxygen (O), and nitrogen (N), confirming the successful capture of AA.

### 3.3. ACEK-Enhanced Point-of-Care Detection System for AA

**Figure 3:**
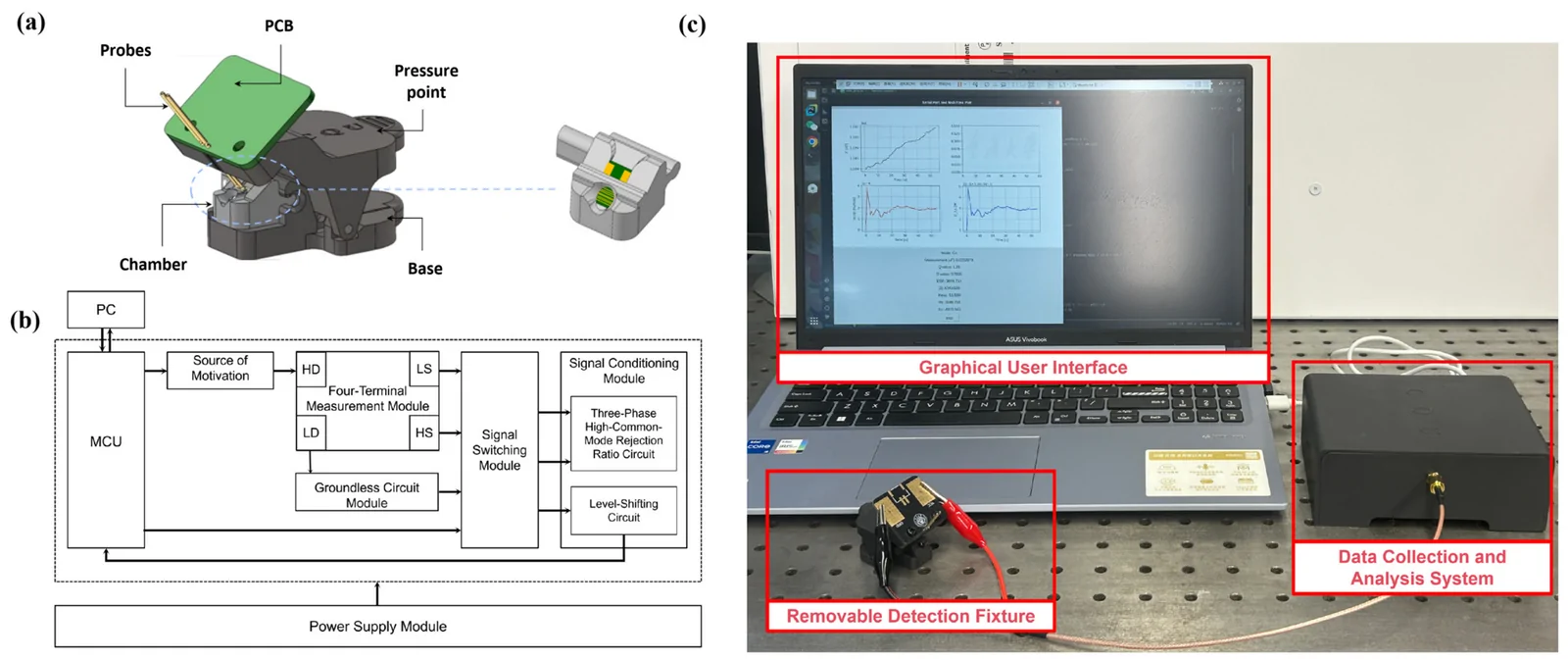
(**a**) SolidWorks assembly drawings of the removable testing fixture and incubation chamber. (**b**) Overall schematic diagram of the sensor data acquisition module. (**c**) Physical diagram of the ACEK-enhanced POCT system.

Figure 3a shows the structure of the test fixture. The incubation chamber for holding the electrodes can be freely removed. The measurement system operates based on the auto-balancing bridge principle, which ensures high-accuracy capacitance detection by exploiting the virtual short-circuit characteristic of an operational amplifier (op-amp) operating in its linear region. The overall system architecture is shown in Figure 3b. The developed acquisition module combines the auto-balancing bridge principle with digital PSD, consisting of several key functional blocks, including a microcontroller unit (MCU) subsystem, excitation source module, four-terminal sensing module, virtual ground circuit, analog signal switching module, signal conditioning module, analog-to-digital conversion (ADC) unit, and power management module.

During system operation, the excitation source module first converts the PWM (pulse width modulation) signal output by the MCU into a high-precision sinusoidal excitation signal, which is then applied to the “HD” terminal of the four-terminal measurement module. The “LD” terminal is connected to the virtual ground circuit, maintaining its potential at ground level. The output signal from the virtual ground circuit is transmitted through the “HS” and “LS” ports to the analog signal switching module. The analog signal switching module dynamically selects the measurement channels, routing the voltage and current signals from both ends to three operational amplifier differential amplifiers with high common-mode rejection ratio (CMRR), respectively. The processed analog signals are then amplified and level-shifted to match the MCU’s ADC input range, thereby ensuring precise digitization. Figure 3c shows a schematic diagram of the detection system. In terms of human–machine interaction, the MCU communicates with a PC via a USB interface to enable real-time data acquisition, visualization, and quantitative analysis of changes in interfacial capacitance, correlating these with AA concentration.

### 3.4. Optimization of ACEK Excitation Parameters

Optimization experiments were conducted using 10 nmol/L AA solution as the model analyte. The normalized capacitance change rate (dC/dt) was employed as the figure of merit to evaluate the impact of excitation amplitude and frequency on AA enrichment efficiency. First, an interdigitated electrode model was constructed using COMSOL Multiphysics 6.1 software (Figure S3), and the AC frequency and amplitude were optimized. Figure S4 shows the evolution of the electric field magnitude distribution within the sensing region as the excitation frequency increases from 1 Hz to 1 MHz while maintaining a fixed amplitude of 100 mV. The results revealed that, at 100 Hz, 1 kHz, and 10 kHz, the electric field magnitude exhibited both high temporal stability and strong driving capability. Figure S5 shows the variation in the electric field vector on the electrode surface as the amplitude of the excitation signal increases from 100 mV to 1000 mV. The results show that the electric field modulus continues to increase as the amplitude of the excitation source increases. However, an excessively high amplitude of the excitation source can affect the activity of the aptamer and reduce the sensitivity of the sensor. Therefore, based on the simulation results, this study conducted further experimental verification to determine the optimal amplitude and frequency parameters of the excitation signal.

**Figure 4:**
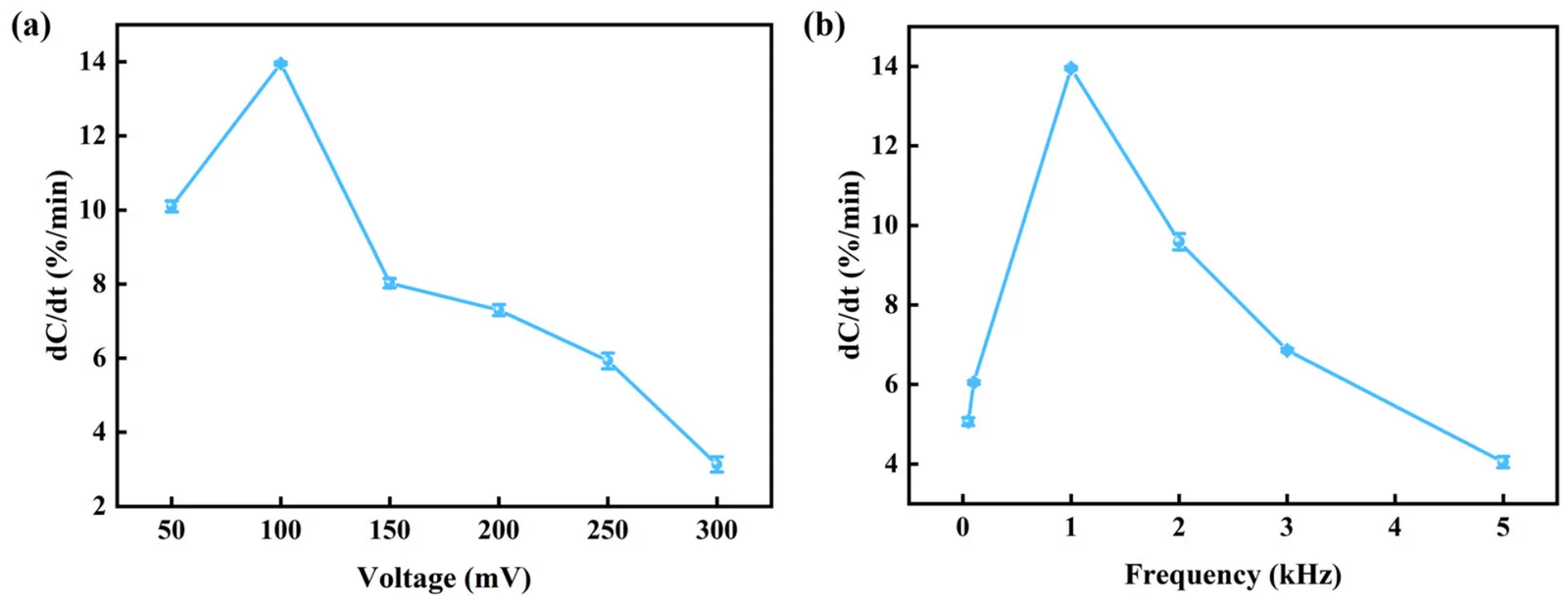
Optimization of detection performance. (**a**) The normalized capacitance change rate of the aptasensor over time when the voltage is 50, 100, 150, 200, 250, and 300 mV. (**b**) The normalized capacitance change rate of the aptasensor over time when the AC signal is 50, 100, 1 k, 2 k, 3 k, and 5 kHz.

In the experiment, the excitation frequency was fixed at 1 kHz, while the amplitude was set to 50 mV, 100 mV, 150 mV, 200 mV, 250 mV, and 300 mV. The normalized rate of capacitance change over 1 min was recorded for 10 nmol/L AA. As shown in Figure 4a, the maximum rate of change was achieved at 100 mV, indicating that the ACEK-induced enrichment effect was optimal at this amplitude. Therefore, 100 mV was selected as the optimal excitation amplitude. Next, frequency optimization was performed by fixing the AC amplitude at 100 mV and evaluating the following frequencies: 50 Hz, 100 Hz, 1 kHz, 2 kHz, 3 kHz, and 5 kHz. The normalized rate of capacitance change for 10 nmol/L AA over 1 min peaked at 1 kHz (Figure 4b), indicating that enrichment efficiency was highest at this frequency. Therefore, 1 kHz was selected as the optimal frequency. By combining COMSOL simulations with experimental validation, the optimal excitation parameters were ultimately determined to be 1 kHz and 100 mV.

### 3.5. Quantitative Detection of AA

**Figure 5:**
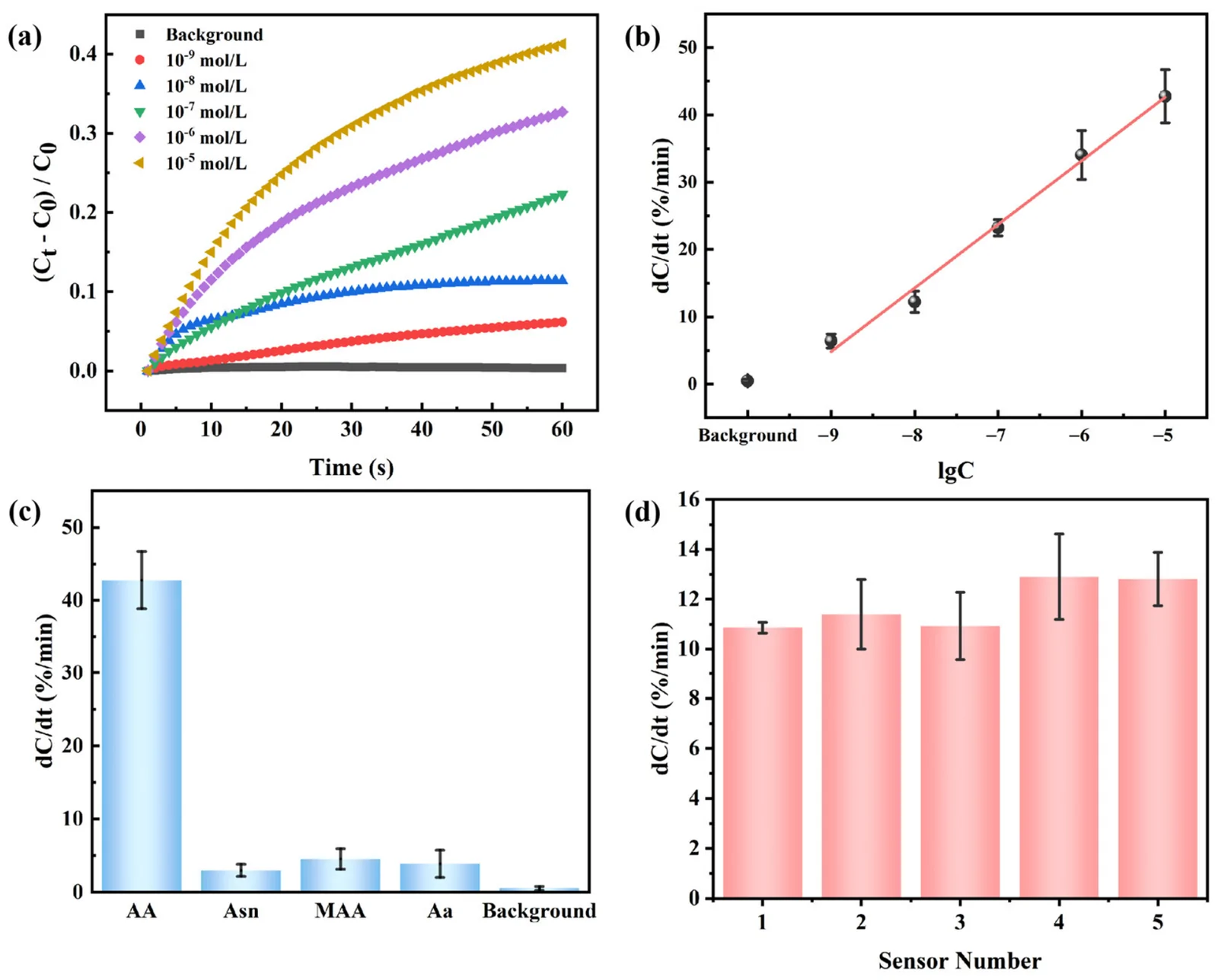
(**a**) Normalized capacitance changes with time for different concentrations of AA over 1 min. (**b**) Fitted curve showing the relationship between the rate of change in normalized capacitance and AA concentration. (**c**) Comparison of the responses to AA and relevant interferents. (**d**) Comparison of the responses of five aptasensors to a concentration of 10 nmol/L AA.

To validate the analytical performance of the ACEK-enhanced capacitive aptasensor, AA standards spanning multiple concentrations were assayed under optimized conditions (100 mV, 1 kHz), with each level tested in quintuplicate (*n* = 5). As shown in Figure 5a, the rate of change in interfacial capacitance increases monotonically with increasing AA concentration. At the end of the 1 min measurement, a clear separation was observed between the different concentration levels. In Figure 5b, the calibration curve is plotted with the logarithm of AA concentration as the independent variable. A robust linear relationship was established in the range from 1 nmol/L to 10 µmol/L. Using ordinary least squares regression, the fitted equation was determined to be y = 9.4532x + 89.9031 (R^2^ = 0.9914). The limit of detection (LOD) was determined using the IUPAC-recommended method, accounting for measurable background signal. The calculated LOD was 0.4235 nmol/L. As shown in Figure 5c, the response rates (R) for all interferents were less than 10%, indicating that the cross-reactivity of the aptamers is negligible. The response value for AA was significantly higher than those for the interferents and the blank group, both of which clustered near the baseline. These results confirm that the aptamers exhibit high specificity for AA and extremely low affinity for structural analogs or precursor compounds. The reproducibility experiment was conducted using a 10 nmol/L AA solution. Five aptasensors were prepared, and five parallel measurements were performed for each sensor to calculate the rate of change in interfacial capacitance over a 1 min period. A uniform standard was applied to the preparation process and data processing for all sensors to ensure consistency in experimental conditions. As shown in Figure 5d, the RSD of the sensor responses across different batches was 7.6363%. There was good consistency among the five sensors, demonstrating that this sensor has good reproducibility.

### 3.6. Application in Real Coffee Samples

Here, the developed aptasensor was applied to quantify AA in liquid coffee, with recovery studies assessing accuracy and robustness. As shown in Table 1, the recovery rates for AA detection in coffee using the sensor developed in this study ranged from 93.96% to 104.63%, with an RSD ≤ 5.42%. This confirms the method’s excellent accuracy and precision in the coffee matrix, as well as its strong resistance to interference and target specificity. Although not exhaustive across all AA-prone foods, the established “pretreatment–capacitive monitoring” framework is highly transferable. Future efforts will extend validation to potato chips, bread, and roasted products using standardized extraction and spike protocols, enabling cross-matrix standardization and broader deployment in food safety screening.

Table 2 compares the present method with previously reported AA detection strategies. This ACEK-enhanced aptasensor exhibits a broader linear range, lower LOD, and shorter assay time. The extended dynamic range and enhanced sensitivity arise from synergistic ACEK-induced aptamer conformational switching and electrokinetic enrichment (via ACET and ACEO forces), while rapid response results from electric field-accelerated mass transport. Relative to chromatographic techniques, the aptasensor offers comparable sensitivity, superior specificity, simplified sample pretreatment, ease of operation, and significantly reduced cost.

## 4. Conclusions

This study developed an ACEK-enhanced capacitive aptasensor for rapid AA detection, culminating in a fully integrated point-of-care system. The platform integrates a high-specificity aptamer interface, optimized electrokinetic enrichment, precision capacitance readout, and user-friendly software, demonstrating robust performance in both standard solutions and complex coffee matrices—particularly excelling in assay speed. The synergistic integration of capacitive aptasensing and ACEK-mediated enrichment enables ultrasensitive, rapid, and matrix-tolerant AA quantification, positioning the platform as a viable tool for on-site food safety screening.

## Figures and Tables

**Table 1:** Quantification of AA in the spiked samples using the designed aptasensor (*n* = 3).

Original Concentration (mol/L)	Spiked Concentration (mol/L)	Measured Concentration(mol/L)	Recovery (%)	RSD (%, *n* = 3)
10^−8^	10^−8^	(1.9999 ± 0.0840) × 10^−8^	96.44~104.63	4.20
10^−7^	10^−7^	(1.9633 ± 0.0642) × 10^−7^	95.58~101.76	3.27
10^−6^	10^−6^	(2.0035 ± 0.1086) × 10^−6^	93.96~103.99	5.42

**Table 2:** The performance comparison among recent aptasensor for AA detection.

Type	Linear Range	LOD	Time	Recovery (%)	Ref.
Colorimetric	0.05~200 nmol/L	0.038 nmol/L	-	92~102	[28]
Fluorescence	0.001~100 μmol/L	1.53 nmol/L	50 min	-	[31]
Electrochemical	0.39~50.0 μg/mL	0.033 μg/mL	30 min	88.29~111.76	[34]
Electrochemical	1~600 nmol/L	0.104 nmol/L	-	98.7~103.4	[12]
Colorimetric	0.05~15 μM	15.4 nM	-	95.3~103.6	[35]
Capacitive	0.001~10 μmol/L	0.4235 nmol/L	1 min	93.96~104.63	This work

## Data Availability

Data are contained within the article and Supplementary Materials.

## Supplementary Materials

The following supporting information can be downloaded at https://www.mdpi.com/article/10.3390/mi17080966/s1. Figure S1: Schematic diagram of AECK-enhanced functionalized electrode surface specific binding. (a) The functionalized electrode surface with no AC signal. (b) The functionalized electrode surface with AC signal. Figure S2: The EDS of (a) MUA/Au, (b) Apt/MUA/Au, and (c) Eth/Apt/MUA/Au. Figure S3: (a) The physical diagram of silicon-based bipolar interdigital gold electrode. (b) The COMSOL modeling diagram of interdigital gold electrode. Figure S4: The COMSOL modeling diagram of the electric field norm at t_1_ and t_2_ with an amplitude of 100 mV and a frequency ranging from 1 Hz to 1 MHz. Figure S5: The COMSOL modeling diagram of the electric field norm at t_1_ and t_2_ with the frequency of 1 kHz with an amplitude ranging from 100 to 1000 mV. Figure S6: Simulation of the ACET and ACEO effects generated by a 100 mV, 1 kHz AC excitation. (a) Temperature distribution generated by a non-uniform alternating electric field in the fluid. (b) Velocity distribution of fluid flow driven by the ACET effect. (c) Velocity distribution of fluid flow driven by the ACEO effect. (d) Streamlines of the concentration gradient in the solution under the coupled ACET and ACEO effects.

## Abbreviations

The following abbreviations are used in this manuscript:

AA	Acrylamide
GC–MS	Gas chromatography–mass spectrometry
LC–MS/MS	Liquid chromatography–tandem mass spectrometry
HPLC	High-performance liquid chromatography
CE	Capillary electrophoresis
ELISA	Enzyme-linked immunosorbent assay
QCM	Quartz crystal microbalance
FRET	Fluorescence resonance energy transfer
Apt	Aptamer
SELEX	Systematic Evolution of Ligands by Exponential Enrichment
POCT	Point-of-care testing
ACEK	alternating current electrokinetics
SAM	Self-assembled monolayer
MUA	11-mercaptoundecanoic acid
RSD	Relative standard deviations
PBS	Phosphate-buffered saline
EDC	1-(3-dimethylaminopropyl)-3-ethylcarbodiimide
PSD	Phase-sensitive detection
SNR	signal-to-noise ratio
ACET	AC Electrothermal
ACEO	AC Electroosmosis
SEM	Scanning electron microscopy
MCU	Microcontroller unit
ADC	Analog-to-digital conversion
PWM	Pulse width modulation
EDS	Energy-dispersive X-ray spectroscopy
Eth	Ethanolamine hydrochloride
