# Supplementary material for: AC Electrokinetics-Enhanced Capacitive Aptasensor for Point-of-Care Testing of Acrylamide in Coffee
Source: Micromachines (Basel). 2026 Aug 16;17(8):966. doi: 10.3390/mi17080966 (PMC13515806; doi:10.3390/mi17080966)
Supplement: Supplementary file 1 [file micromachines-17-00966-s001.zip › micromachines-4446638-supplementary.pdf]

The Supporting Information for

AC electrokinetics-enhanced capacitive aptasensor for point-of-care testing of Acrylamide in coffee

Ke Wang [1], Mingna Xie [1], Yuyang Zhao [1], Jiuyi Wang [1], Leilei Zeng [1], Xiaogang Lin [1,*] and Jie Jayne Wu [2]

1 Key Laboratory of Optoelectronic Technology and Systems of Ministry of Education of China, Chongqing University, Chongqing 400044, China; cqu_wke@163.com (K.W.); mingnaxie2017cqu@163.com (M.X.); 202508131179@stu.cqu.edu.cn (Y.Z.); 202408021039t@stu.cqu.edu.cn (J.W.); zengll0310@163.com (L.Z.)

2 Department of Electrical Engineering and Computer Science, The University of Tennessee, Knoxville, TN 37996, USA; jaynewu@utk.edu (J.W.)

* Correspondence: xglin@cqu.edu.cn (X.L.)

1. The Mechanism of ACEK Effect Enhancement

In capacitive sensors, the electric double layer (EDL) can be regarded as the dielectric layer of an interfacial capacitor; when macromolecules adsorb onto the electrode surface, the thickness of the dielectric layer increases, causing a change in the interfacial capacitance. Figure S1 shows the interfacial changes in a capacitive aptamer sensor based on the ACEK effect. Under conditions where a specific AC signal is applied, the microscopic eddy currents generated by the ACEK act on the surface of the functionalized interdigitated electrodes, accelerating the enrichment process of the

target analyte, thereby enhancing sensor performance and shortening the detection time. Both the AC electrothermal (ACET) and AC electroosmotic (ACEO) effects act on the bulk solution and promote the migration of the target analyte toward the surface of the aptamer-functionalized electrodes. The ACET effect is not limited to the double layer, whereas the ACEO effect primarily depends on the dispersion layer of the double layer and is influenced by its thickness. In this study, ultrapure water (conductivity approximately 0.05 – 0.055 μS/m) was selected as the background solution; when the electrode was exposed to a non-uniform AC electric field, the effects of ACET and ACEO were particularly pronounced. On the other hand, the dielectrophoresis (DEP) effect primarily targets particles with a polarizability higher than that of the medium. However, since AA is an organic small-molecule compound with a polarizability similar to that of the background solution, the contribution of dielectrophoretic forces to AA enrichment is relatively small within the scope of this study.

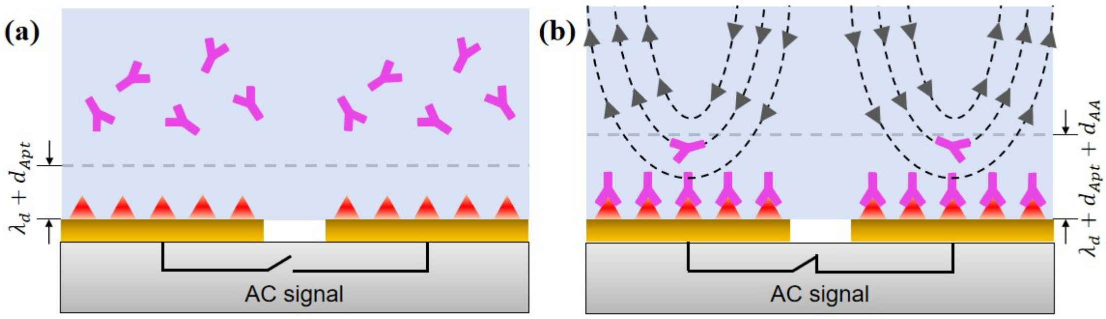

Figure S1. Schematic diagram of AECK-enhanced functionalized electrode surface specific binding. (a) The functionalized electrode surface with no AC signal. (b) The functionalized electrode surface with AC signal.

2. EDS of the Electrode Functionalization

To evaluate changes in the elemental composition of the electrode surface during the stepwise functionalization process, energy-dispersive X-ray spectroscopy (EDS) analysis was employed to determine the elemental composition and verify the effectiveness of the modification. Figure S2 shows the results of EDS of MUA/Au, Apt/MUA/Au, and Eth/Apt/MUA/Au.

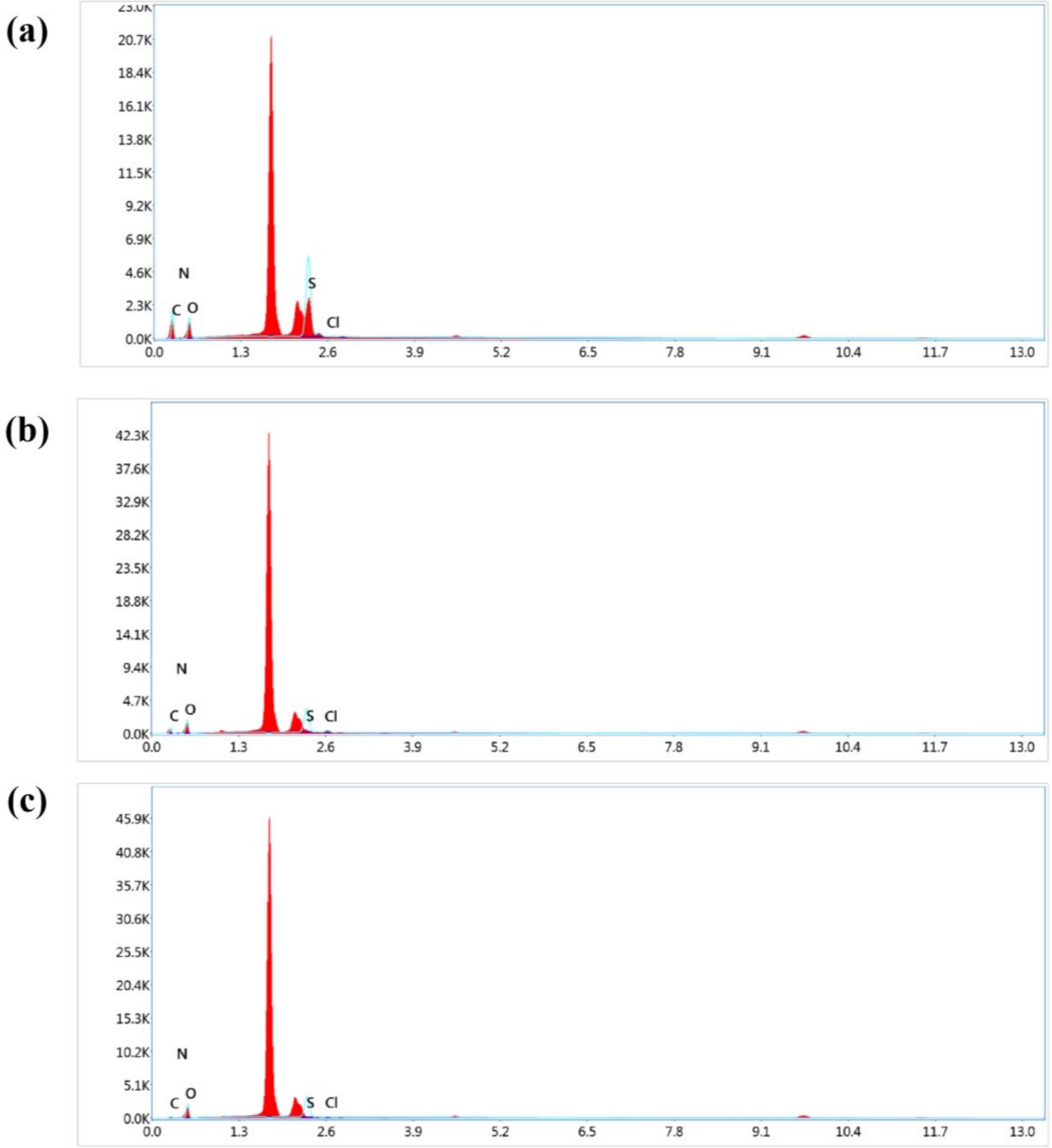

Figure S2. The EDS of (a) MUA/Au, (b) Apt/MUA/Au, and (c) Eth/Apt/MUA/Au.

3. Simulation and Optimization of AC Frequency and Amplitude

In this study, based on the geometric parameters of the selected interdigitated electrodes, simulation modeling was performed using COMSOL finite element software (Figure S3). By adjusting the amplitude and frequency of the excitation signal, the magnitude of the external electric field around the interdigitated electrodes was observed, and this data was used to optimize the response characteristics of the interfacial capacitance.

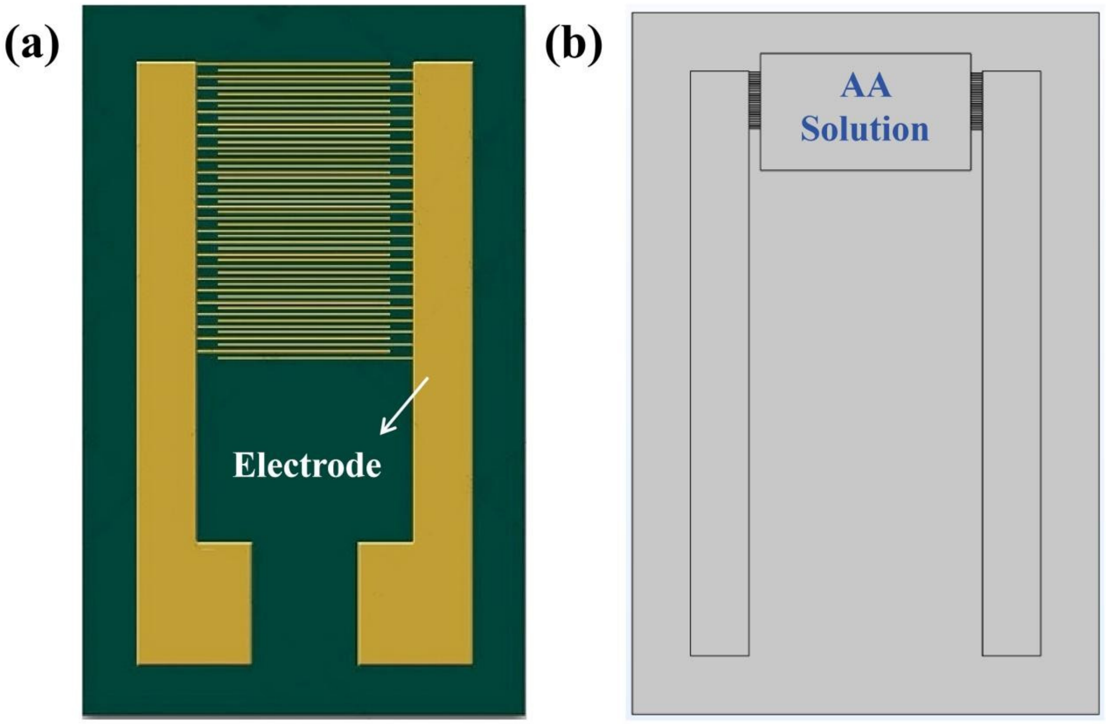

Figure S3. (a) The physical diagram of silicon-based bipolar interdigital gold electrode. (b) The COMSOL modeling diagram of interdigital gold electrode.

In the simulation analysis, the amplitude of the excitation source was first fixed at 100 mV to investigate the distribution of the electric field norm on the electrode surface as the frequency varied from 1 Hz to 1 MHz (Figure S4). Additionally, to further analyze the time dependence of the electric field under an AC voltage, the electric field modulus

was observed at one-quarter (t_1) and one-half (t_2) of the excitation signal period. The electric field norm exhibits high temporal stability and strong driving capability under conditions of 100 Hz, 1 kHz, and 10 kHz. Considering that the low-frequency range (0.1 Hz to 1 kHz) is suitable for studying double-layer capacitance characteristics, while the high-frequency range (1 kHz to 1 MHz) is more suitable for analyzing electron or ion transport behavior at the solid－liquid interface, this study preliminarily selected 1 kHz as the target frequency for the optimization experiment and further investigated the effect of the excitation source amplitude on the interfacial capacitance response.

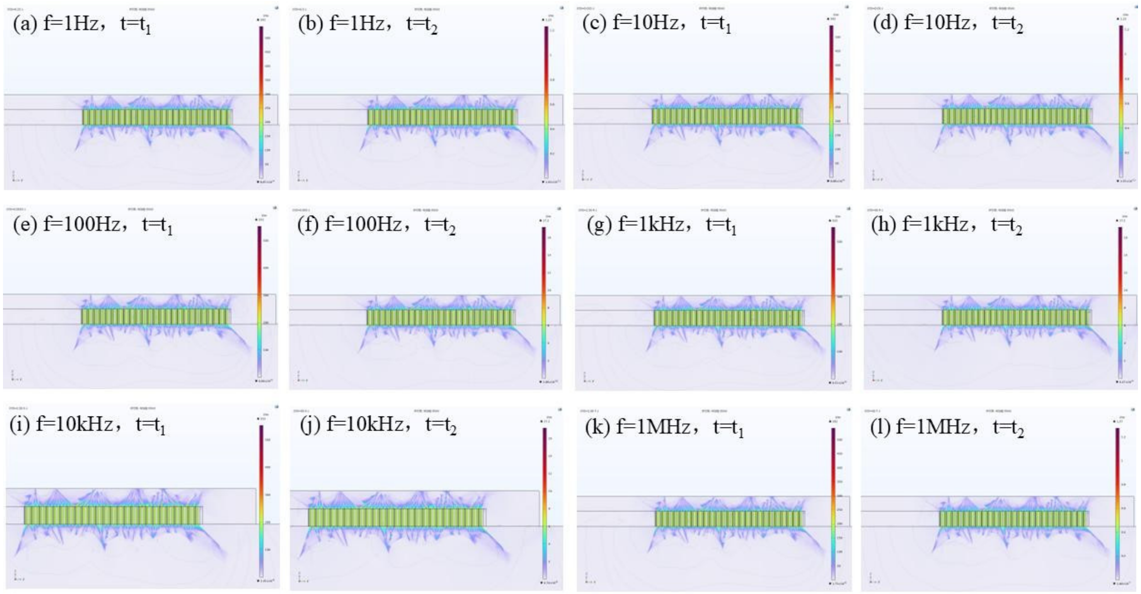

Figure S4. The COMSOL modeling diagram of the electric field norm at t_1 and t_2 with an amplitude of 100 mV and a frequency ranging from 1 Hz to 1 MHz.

Next, the AC frequency was fixed at 1 kHz to investigate how the electric field norm at the electrode surface changed as the amplitude of the excitation signal increased from 100 mV to 1000 mV (Figure S5). The results showed that the electric field norm continued to increase as the amplitude of the excitation source increased. However, an excessively high excitation amplitude can affect the activity of the aptamer and reduce

the sensitivity of the sensor. Therefore, based on the simulation results, this study conducted further experimental validation to determine the optimal amplitude and frequency parameters for the excitation signal.

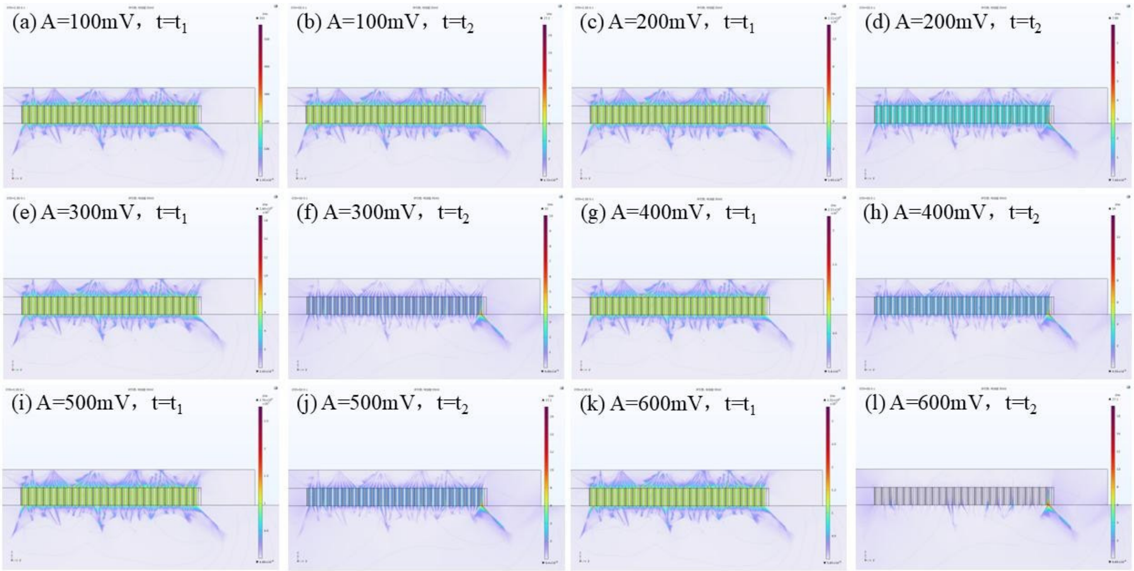

Figure S5. The COMSOL modeling diagram of the electric field norm at t_1 and t_2 with the frequency of 1 kHz with an amplitude ranging from 100 to 1000 mV.

4. COMSOL Simulations of the ACET and ACEO Effects

To verify the ACET and ACEO effects as the dominant mechanisms of the ACEK effect, we constructed a two-dimensional model of a pair of interdigitated gold electrodes in COMSOL, setting the solution medium to 1×PBS and the AA concentration to 10 nmol/L. By applying a 100 mV, 1 kHz AC signal to the electrodes, we investigated the distribution of fluid temperature induced by the non-uniform electric field, as well as the ACET and ACEO flow velocities and the solution concentration gradient. The visualization results of the specific simulations are shown in Figure S6.

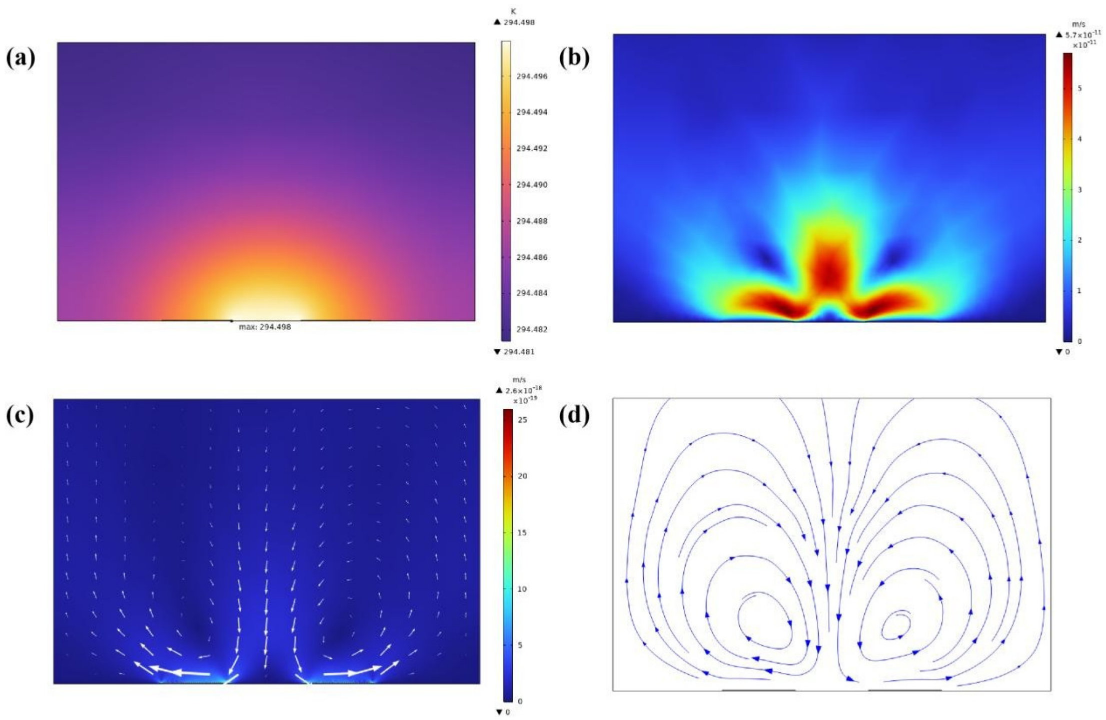

Figure S6. Simulation of the ACET and ACEO effects generated by a 100 mV, 1 kHz AC excitation. (a) Temperature distribution generated by a non-uniform alternating electric field in the fluid. (b) Velocity distribution of fluid flow driven by the ACET effect. (c) Velocity distribution of fluid flow driven by the ACEO effect. (d) Streamlines of the concentration gradient in the solution under the coupled ACET and ACEO effects.
